# A population-specific material model for sagittal craniosynostosis to predict surgical shape outcomes

**DOI:** 10.1007/s10237-019-01229-y

**Published:** 2019-09-30

**Authors:** Alessandro Borghi, Naiara Rodriguez Florez, Federica Ruggiero, Greg James, Justine O’Hara, Juling Ong, Owase Jeelani, David Dunaway, Silvia Schievano

**Affiliations:** 1 UCL Great Ormond Street Institute of Child Health and Great Ormond Street Hospital for Children, London, UK; 2 Surface Technologies Group, Department of Biomedical Engineering, Mondragon Unibertsitatea, Mondragón, Spain

**Keywords:** Craniofacial surgery, Scaphocephaly, Spring cranioplasty, Finite element modelling, Design of experiments

## Abstract

Sagittal craniosynostosis consists of premature fusion (ossification) of the sagittal suture during infancy, resulting in head deformity and brain growth restriction. Spring-assisted cranioplasty (SAC) entails skull incisions to free the fused suture and insertion of two springs (metallic distractors) to promote cranial reshaping. Although safe and effective, SAC outcomes remain uncertain. We aimed hereby to obtain and validate a skull material model for SAC outcome prediction. Computed tomography data relative to 18 patients were processed to simulate surgical cuts and spring location. A rescaling model for age matching was created using retrospective data and validated. Design of experiments was used to assess the effect of different material property parameters on the model output. Subsequent material optimization—using retrospective clinical spring measurements—was performed for nine patients. A population-derived material model was obtained and applied to the whole population. Results showed that bone Young’s modulus and relaxation modulus had the largest effect on the model predictions: the use of the population-derived material model had a negligible effect on improving the prediction of on-table opening while significantly improved the prediction of spring kinematics at follow-up. The model was validated using on-table 3D scans for nine patients: the predicted head shape approximated within 2 mm the 3D scan model in 80% of the surface points, in 8 out of 9 patients. The accuracy and reliability of the developed computational model of SAC were increased using population data: this tool is now ready for prospective clinical application.

## Introduction

The infant cranial vault consists of flat bones joined by cranial sutures (Opperman 2000), membranous soft tissue important both at birth and for brain growth. Sutures close naturally over time; however, premature suture closure—called craniosynostosis—is a pathology with a prevalence of up to one in 1700 live births (Fearon 2014). Newborns affected by premature closure of the sagittal suture (sagittal craniosynostosis—SC) develop an elongated and narrow head because of compensatory growth of the skull in the direction parallel to the affected suture (Garza and Khosla 2012; O’Hara et al. 2019). Although this pathology mainly causes aesthetic problems, recent findings show that up to 24% of patients affected by non-syndromic craniosynostosis develop also intracranial hypertension (Wall et al. 2014) with cognitive, speech and behavioural sequelae.

SC appears to be the most frequent isolated synostosis. A number of procedures have been described to treat sagittal synostosis with no clear consensus on timing and technique. The range of methods goes from total calvarial remodelling, when the surgeon harvests and repositions several bone flaps in the whole vault, to minimally invasive procedures (i.e. spring-assisted cranioplasty, endoscopically assisted suturectomy). Modified and standard pi procedures, alongside with barrel staving biparietal expansion, have been described too. The attractiveness of minimally invasive techniques comes mainly from the reduced surgical access needed, the lower rate of blood transfusion and the shorter operating and hospitalization time. In our Centre, the preferred minimally invasive technique is spring-assisted cranioplasty (SAC), which allows the treatment of scaphocephaly at an earlier stage in life (Windh et al. 2008; Greensmith et al. 2008; Taylor and Maugans 2011; Zakhary et al. 2014).

**Fig. 1:**
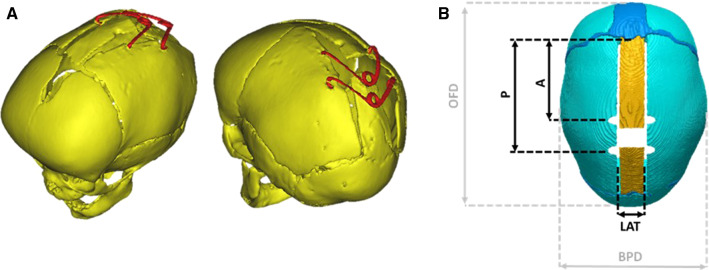
**a** 3D reconstruction of post-operative CT images from a SAC patient; **b** Surgical osteotomies and measurements recorded during surgery: *A* = distance between the coronal suture and the anterior spring; *P* = distance between the coronal suture and the posterior spring; *LAT* = dimension of the parasagittal osteotomy; *OFD* = occipitofrontal diameter; *BPD* = biparietal diameter

SAC was first introduced in 1998 by Lauritzen et al. (1998) as an alternative to more invasive cranial vault remodeling surgeries (Ocampo and Persing 1994). During SAC, minimal skull osteotomies are performed in order to release the synostosed suture (Rodgers et al. 2017) and springs are temporarily inserted to drive calvarial augmentation (Fig. 1a). At Great Ormond Street Hospital for Children (GOSH), London, SAC has been routinely adopted for the treatment of SC patients aged 3–6 months since 2008 (Rodgers et al. 2017). Our group has investigated the dynamic nature of SAC by measuring spring opening over time (Borghi et al. 2017b), 3D head shape adaptation results investigated through 3D scanning (Tenhagen et al. 2016) and the association between surgical parameters and head shape feature outcomes via statistical shape modelling (Rodriguez-Florez et al. 2017). In addition, we presented a SAC case study using patient-specific finite element (FE) modelling, which proved that the overall head shape change can be realistically modelled, provided the correct location of springs as well as spring models is recorded during surgery: the material properties needed patient-specific tuning to accurately capture spring/skull behaviour over time (Borghi et al. 2018). As stated by Malde et al. (2019), only a few works in the literature describe methods for predicting the surgical outcome of craniosynostosis correction. Works in the literature have proven that numerical modelling of craniosynostosis repair can help perform surgical osteotomy planning (Larysz et al. 2012; Wolański et al. 2013; Li et al. 2017) as well as drive the selection of the distraction devices (Zhang et al. 2016; Borghi et al. 2018). Simulation results provide information on the sensitivity of the surgical outcomes of pathological severity (Nagasao et al. 2011) and bone characteristics (Li et al. 2017) as well as performance of difference surgical strategies (Wolański et al. 2013).

Data from the literature relative to bone mechanical properties in paediatric subjects aged 3–8 months show high dispersion, with values of Young’s modulus ranging from 186 to 3800 MPa (Thibault et al. 1999; Margulies 2000; Coats and Margulies 2006; Li et al. 2011); furthermore, bone mechanical properties are highly affected in pathological conditions (Carriero et al. 2014; Imbert et al. 2015). Therefore, in this work, we hypothesize that the overall behaviour of the scaphocephalic head subject to spring distractors would be better evaluated by means of population-based material properties. A similar assumption was adopted by Bosi et al. (2018) who used a routine design engineering approach to investigate the effect of material parameters in transcatheter aortic valve implantation modelling and tuned a population-based material model which improved the prediction of post-intervention device performance. Our group has in the past investigated the use of population-specific material properties to improve and assess statistical variations in orthognatic surgery prediction using retrospective data from a historical cohort (Knoops et al. 2018).

In this work, an FE framework for the simulation of SAC was created by applying the previously developed methodology to a patient cohort and by quantifying the accuracy of the methodology comparing the simulated spring expansion with actual patient measurements.

We hypothesize that the use of population-tuned material properties would allow for better prediction of cranial reshaping dynamics compared to material models available in the literature. The calvarial material parameters were tuned by means of design of experiment (DoE) as well as response surface optimization in a subpopulation, in order to derive a population specific set of material parameters.

## Methodology

Patients who received pre-operative CT scans before undergoing SAC were recruited. CT scans were exported from the GOSH electronic database; information relative to osteotomy (surgical cuts) size and location as well as on-table spring performance were retrieved during surgery (by means of a sterilized ruler). FE models of patient surgeries were created based on pre-operative CT scans, on-table surgical data, spring openings measured from follow-up X-rays and literature data for the material model. In a second step, material parameters were optimized using a subset of the population and the tuned data re-entered in the FE model of all patients to assess if the prediction of the spring performance improved. Surface scan data, available for a subset of patients at the time of surgery, just after spring insertion, allowed comparison with the overall on-table resulting head shape from the simulations.

### Patient population

Eighteen SC patients (all male, age at surgery = 5.5 ± 1.0 months, weight at surgery 7.6 ± 1.0 kg) who underwent SAC at GOSH between March 2014 and December 2017 were retrospectively recruited for this study (Table 1). All patients had pre-operative computed tomography (CT) scans (age at scan = 3.7 ± 1.5 months). SAC requires two osteotomies parallel to the fused suture, at a distance LAT extending from the coronal to the lambdoid sutures (Fig. 1b). A craniectomy is performed to allow for lateral expansion and the piece of bone is discarded. Two spring distractors are then inserted, one anteriorly (at a distance A from the coronal suture) and one posteriorly (at a distance *P* from the coronal suture, Fig. 1b). Further clinical details about the SAC procedure can be found in previous publications from our group (Borghi et al. 2017a, 2018; Rodgers et al. 2017).

**Table 1:** Patient population characteristics

Patient #	Age at CT (months)	Age at SAC (months)	Anterior spring	Posterior spring
1	3.7	5.3	S12	S12
2	5.2	5.9	S12	S12
3	7.2	8.0	S14	S14
4	1.6	3.7	S12	S12
5	3.8	6.1	S14	S14
6	3.9	5.0	S14	S14
7	1.8	4.3	S14	S12
8	3.0	4.8	S12	S12
9	5.1	5.5	S14	S14
10	3.2	5.6	S12	S12
11	4.3	5.5	S12	S10
12	4.7	5.4	S14	S14
13	3.7	5.5	S12	S12
14	3.9	4.6	S12	S12
15	1.7	5.2	S12	S12
16	1.4	5.3	S12	S12
17	3.2	6.4	S12	S12
18	5.2	7.5	S14	S14

Osteotomy dimensions (LAT, Fig. 1b), as well as spring model (Table 1), position (anterior *A* and posterior *P* spring) and on-table opening at insertion (OP_I_^M^) were recorded in theatre during surgery (Table 1). Spring expansion at follow-up was measured from planar X-rays acquired 1 day (OP_FU1_^M^) and 1 month (OP_FU2_^M^; 28 ± 12 days) after surgery. A previously validated method of geometric correction for out-of-plane error of X-ray measurements was used here, similarly to other works from our group (Borghi et al. 2017a, 2018).

On-table 3D skin surface scans (*n* = 9, Tenhagen et al. 2016), acquired pre- and post-operatively were, respectively, used for validation of the rescaling method, and for the overall on-table resulting calvarial shape after simulated spring insertion, as further described below.

### Patient-specific FE geometry

**Fig. 2:**
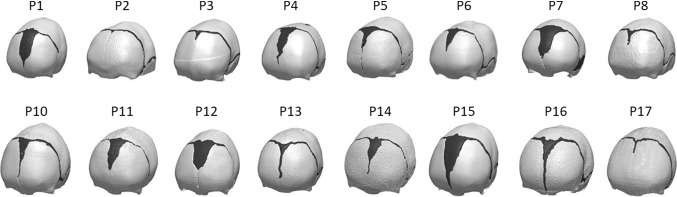
Patient population for the study—each model is made of skull (white) and cranial sutures (black)

3D reconstructions of each patient skull bone and suture anatomy were created by processing the pre-op CT images in SCANIP^®^ (Synopsis, Mountain View, CA) (Fig. 2). Each skull reconstruction was cut with a plane encompassing skeletal nasion, and left and right auditory meatus.

**Fig. 3:**
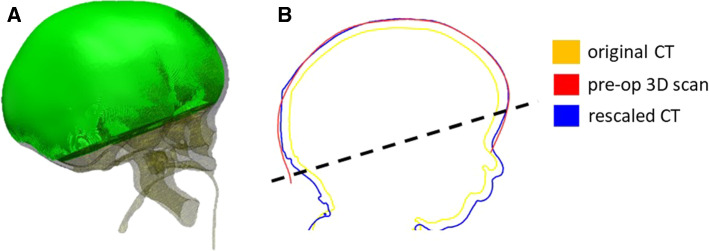
**a** Visualization of the control volume VCT used for the model rescaling. **b** Sagittal cross section of a patient head to show comparison showing comparison between head shape retrieved from the original CT (yellow), the rescaled dataset (blue) and a pre-op 3D scan (red); the region used for the RMSE is above the dotted line

To account for the patient head growth between the time of CT scan and procedure, a population growth curve was created based on the bone surface of 24 unoperated SC patients (22 male, age at scan 4.0 ± 1.3 months). A control volume *V*_CT_ was extracted from these patient CT scans defined by the outer shell of the reconstructed skull—sutures and cranial defects were filled using Meshmixer (Autodesk Inc., San Rafael, CA)—and by a cutting plane parallel to that defined by nasion, left and right auditory meatus and passing through the supraorbital notch (Fig. 3a), in order to avoid eye sockets and additional soft tissue artefacts. A logarithmic growth curve model was extrapolated from the *V*_CT_/age plot:

$$V_{\text{CT}} = a + b*\log \left( {{\text{age}}_{\text{CT}} + 1} \right)$$

where age_CT_ is the age of the patient at the time of the CT, *a* and *b* are parameters defining the logarithmic growth (Breakey et al. 2018). This equation was used to rescale the SAC patient head volumes to the time of spring insertion (age_SAC_):

$$V_{\text{SAC}} = V_{\text{CT}} + b*\log \frac{{1 + {\text{age}}_{\text{SAC}} }}{{1 + {\text{age}}_{\text{CT}} }}$$

For those nine patients who had on-table surface scans available, the skin surface was also reconstructed from the pre-operative CT data and scaled accordingly in order to validate this process (Fig. 3b). For each patient, on-table 3D surface scan and rescaled head model from CT were registered using iterative closest point (ICP) algorithm. The root mean square error (RMSE) of the surface distance between the two shapes was calculated.

Each skull geometry was meshed using linear tetrahedral elements using Simpleware ScanIP, the same mesh density coarseness values were used for each model, and visual inspection ensured that at least three elements were present through the skull thickness of each case. An optimal trade-off between accuracy, i.e. convergence of simulated spring expansion, and CPU time was adopted to select mesh density: the models resulted in an average of 106,636 nodes and 361,225 elements. Simulations took 2.5 h on average to run.

**Fig. 4:**
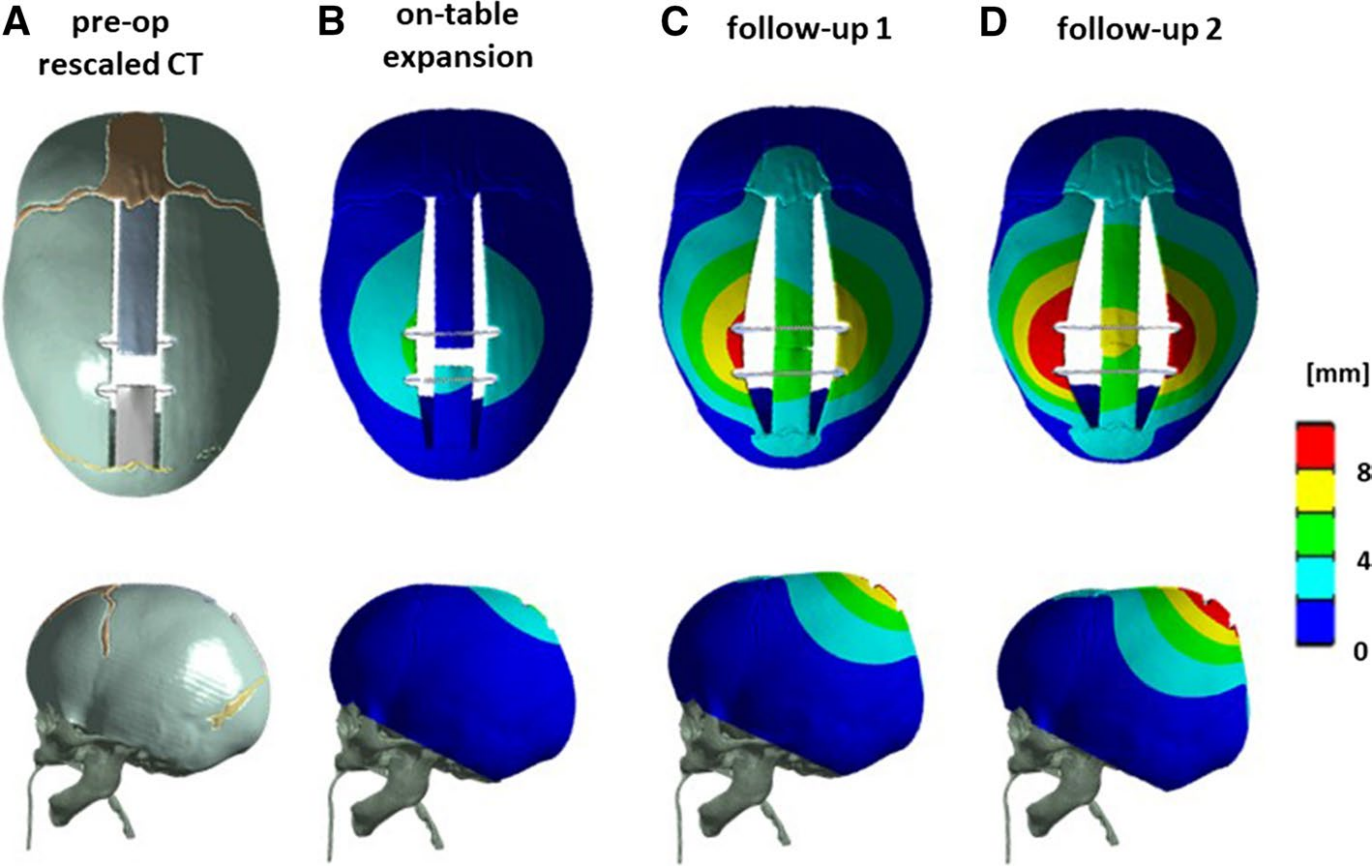
**a** Pre-operative CAD model with spring conditions, **b** simulated spring expansion on-table, **c** at follow-up 1, and **d** 2 for a representative patient—top view (top) and lateral view (bottom)

Each mesh was imported into ANSYS mechanical 17.2 (Canonsburg, Pennsylvania, US). The base of the model was fully constrained to replicate the presence of the calvarial skull base. Surgical cuts (Fig. 4a) were replicated using the patient-specific osteotomy dimensions retrieved from surgery (Borghi et al. 2018). The effect of spring implantation was simulated using linear spring conditions implemented in ANSYS (Borghi et al. 2017a): linear spring conditions were applied to nodes in opposite groves; experimental stiffness and unloaded spring length were published (Borghi et al. 2017a).”

Cranial Index, which is defined as the ratio between the occipitofrontal dimension (OFD) and the biparietal dimension (BPD, Fig. 1b), was used to quantify the change in head shape as predicted by the finite element model.

### FE material optimization

As in a previous work from our group (Borghi et al. 2018), a viscoelastic material model was adopted to mimic the skull reshaping both on-table and over time, due to skull-spring interaction (Davis 2010; Zhang et al. 2016; Borghi et al. 2017a):

$$\frac{G\left( t \right)}{{G_{0} }} = \alpha_{\infty } + \mathop \sum \limits_{i} \alpha_{i} {\text{e}}^{{ - \frac{t}{{\tau_{i} }}}}$$

where *α*_∞_ and *α*_*i*_ are the relative moduli, *τ*_*i*_ are the time constants, *G*(*t*) is the instantaneous shear modulus and *G*_0_ is the shear modulus at the beginning of the relaxation (*t* = 0). A bone relaxation modulus scale constant was introduced to account for different relaxation properties of the pediatric bone, compared to adult calvarium:

$$\alpha_{i}^{\prime} = \alpha_{G} \cdot \alpha_{i}$$

Similarly, a, relaxation time scale *α*_*τ*_ was introduced, to account for different relaxation kinematics:

$$\tau_{i}^{\prime} = \alpha_{G} \cdot \tau_{i}$$

Data of bone and suture elastic and viscoelastic properties (skull Young’s modulus *E*_*B*_, suture Young’s modulus *E*_*s*_, *α*_*τ*_ and *α*_*G*_) were initially retrieved from literature (Li et al. 2011, 2013; Yan and Pangestu 2011).

With this set of values (Table 2), a first batch of simulations was performed for all 18 patients (Fig. 3b–d). Anterior and posterior spring openings resulting from the simulations were averaged for each patient (insertion OP_IO_^R^, follow-up 1 OP_FU1_^R^, follow-up 2 OP_FU2_^R^).

**Table 2:** Material parameters

	DoE material property range	SAC literature	SAC optimized
*E*_*B*_ (MPa)	186–1317 (Thibault et al. 1999; Coats and Margulies 2006; Wang et al. 2014)	421	418 ± 217
*E*_*s*_ (MPa)	8–30 (Coats and Margulies 2006; Li et al. 2011, 2013; Moazen et al. 2015)	16	18 ± 5
*α*_*τ*_	1–21 (Lakes et al. 1979; Yue et al. 2008; Yan and Pangestu 2011)	1	9.45 ± 3.96
*α*_*G*_	1–1.2 (Yan and Pangestu 2011)	1	1.17 ± 0.02

In a subset of patients (*n* = 9), response surface optimization (implemented in ANSYS 17.2) was carried out to assess the parametric correlation between simulated average spring expansions and input material parameters (*E*_*B*_, *E*_*s*_, *α*_*τ*_, *α*_*G*_): DoE was performed by varying each parameter between a lower bound and a upper bound (Table 2) using the ANSYS central composite design algorithm to produce parameter combinations by minimizing the number of simulations necessary to assess the overall trends of the model. Local sensitivity charts (which allow to assess the impact of continuous input parameters on output parameters) for each input parameter were extracted: the software calculates the change of outputs based on the change of inputs independently, when all the other parameters are set to a specific value (ANSYS 17.2 user manual). The local sensitivity of each parameter was extracted, while all the others were maintained fixed at their respective baseline values; each local sensitivity was then averaged for all nine patients.

For each patient, the DoE results were used to perform patient-specific optimization of the model using the spring opening values retrieved at insertion and follow-up (OP_IO_^M^, OP_FU1_^M^, OP_FU2_^M^) as target. For each of the nine patients, the best parameter combination minimizing the overall difference between the simulation results and the target values was calculated. A population average parameter combination was derived by averaging each parameter within the nine patient group.

A final set of simulations was run on all 18 patients with the optimized average set of material parameters to compare the spring opening results after material optimization (OP_IO_^OPT^, OP_FU1_^OPT^, OP_FU2_^OPT^) with the outcomes from literature reference values (OP_IO_^R^, OP_FU1_^R^, OP_FU2_^R^) as well as actual measurements (OP_IO_^M^, OP_FU1_^M^, OP_FU2_^M^).

Spring opening values retrieved from each simulation were normalized to the nominal value of maximum spring opening (60 mm) and expressed as percentage.

### Shape prediction validation

Overall resulting on-table calvarial shape was validated with the surface scan of the nine available patients (Borghi et al. 2018): the simulated post-implantation 3D skull shape of each patient (from the optimized model) was offset by a patient-specific fixed amount (measured from CT) to recreate the scalp surface and compared with post-operative 3D scans after processing both shapes with the same protocol used for the pre-operative 3D scans. RMSE was used to assess the accuracy of the model together with the CI. Furthermore, the deformed skull of each patient was extracted from the simulations and the CI was calculated at the three-time-point (PRE, FU1, FU2).

## Results

### Pre-operative CT reconstruction rescaling

**Fig. 5:**
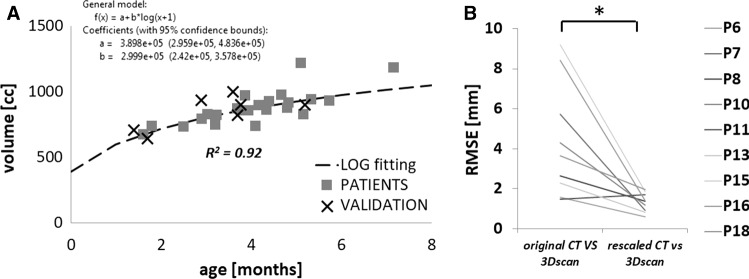
**a** Calvarial growth curve for sagittal craniosynostosis population (squares) with validation points (cross). **b** RMSE between the three head shape models showing accuracy of rescaling (* indicates statistical difference)

In the 18 analysed patients, the average difference between time of CT scan and surgery was 1.8 ± 1.0 months (Table 1). The supraorbital calvarial volume measured for the 24 unoperated patients ranged from 640 mm^3^ (age 1.7 months) to 1,184 mm^3^ (age 7.2 months), and closely followed a logarithmic growth (*R*^2^ = 92.2%, Fig. 5a). The pre-op surface scans available for the nine patients were compared with the initial model reconstructed from CT and with the rescaled model using the growth curve and derived equation: the rescaled model showed statistically significant improvement in RMSE (from 4.37 ± 2.86 to 1.32 ± 1.48 mm, *p* < 0.01, Fig. 5b).

### FE material optimization

**Fig. 6:**
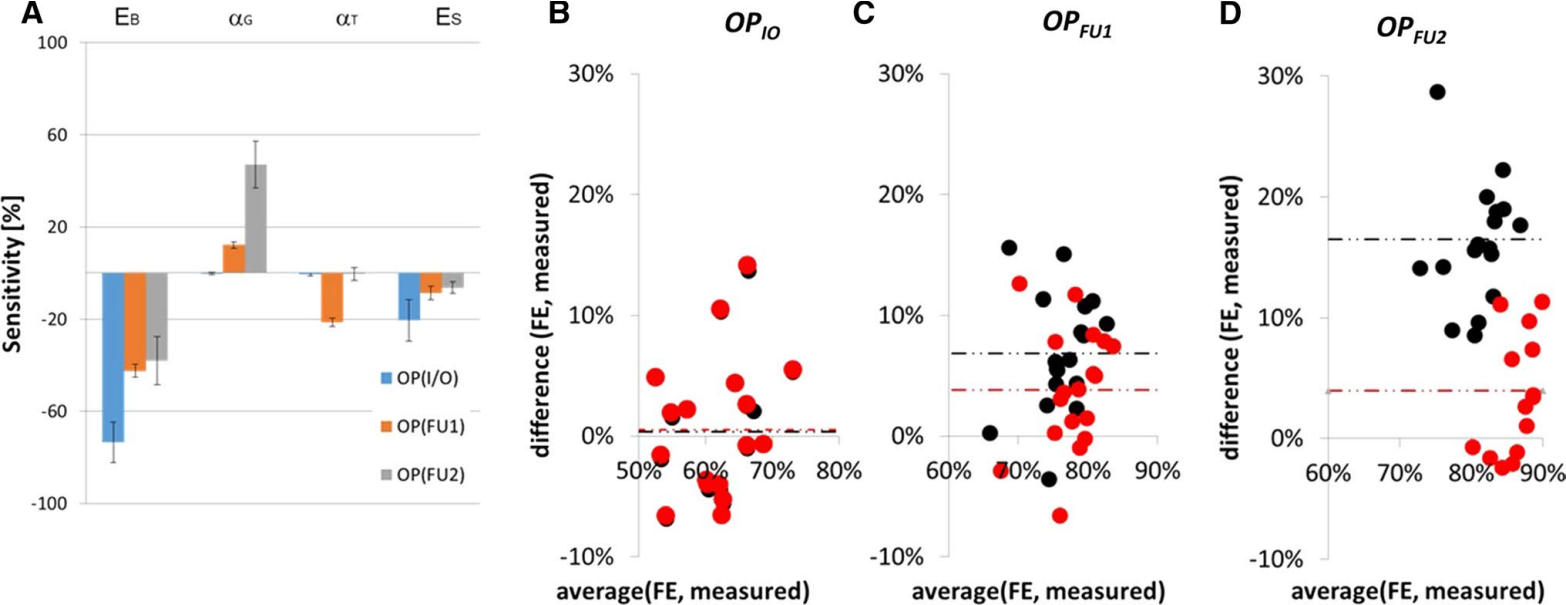
**a** Model sensitivity histogram from the DoE performed on nine patients. Bland–Altman plot showing a comparison between the results of simulations vs measurements in case of literature values of material properties (black) and population optimized material model (red) for **b** insertion (OP_IO_), **c** follow-up 1 (OP_FU1_), **d** follow-up 2 (OP_FU2_); results are shown in terms of percentage of nominal spring size (60 mm)

The DoE performed on the nine patients (Fig. 6a) showed that on-table opening OP_IO_ is mainly affected by bone Young’s modulus (sensitivity − 73.5 ± 8.9%), while follow-up (OP_FU1_ and OP_FU2_) spring performance also depends on relaxation time scale (OP_FU1_: − 21.3 ± 1.9%) and bone relaxation modulus (OP_FU2_: 47.1 ± 10.1%). Suture’s stiffness has a lower impact compared to bone stiffness, more pronounced in the on-table opening (− 20.5 ± 8.9%) and less at follow-ups (OP_FU1_: − 8.7 ± 2.8%, OP_FU2_: − 6.4 ± 2.5%). Table 2 reports the DoE optimized-population-specific material parameters for SAC simulation.

The implementation of population averaged values into the model showed no improvement in the prediction of on-table opening (prediction error: 0.3 ± 5.9% vs. 0.5 ± 5.8%, Fig. 6b), while the prediction of spring opening at FU1 (6.9 ± 5.0% vs. 3.8 ± 5.0%, *p* < 0.01—Fig. 6c) and at FU2 (16.5 ± 4.9% vs. 4.0 ± 5.0%, *p* ≪ 0.01—Fig. 6d) improved significantly.

### FE model validation

**Fig. 7:**
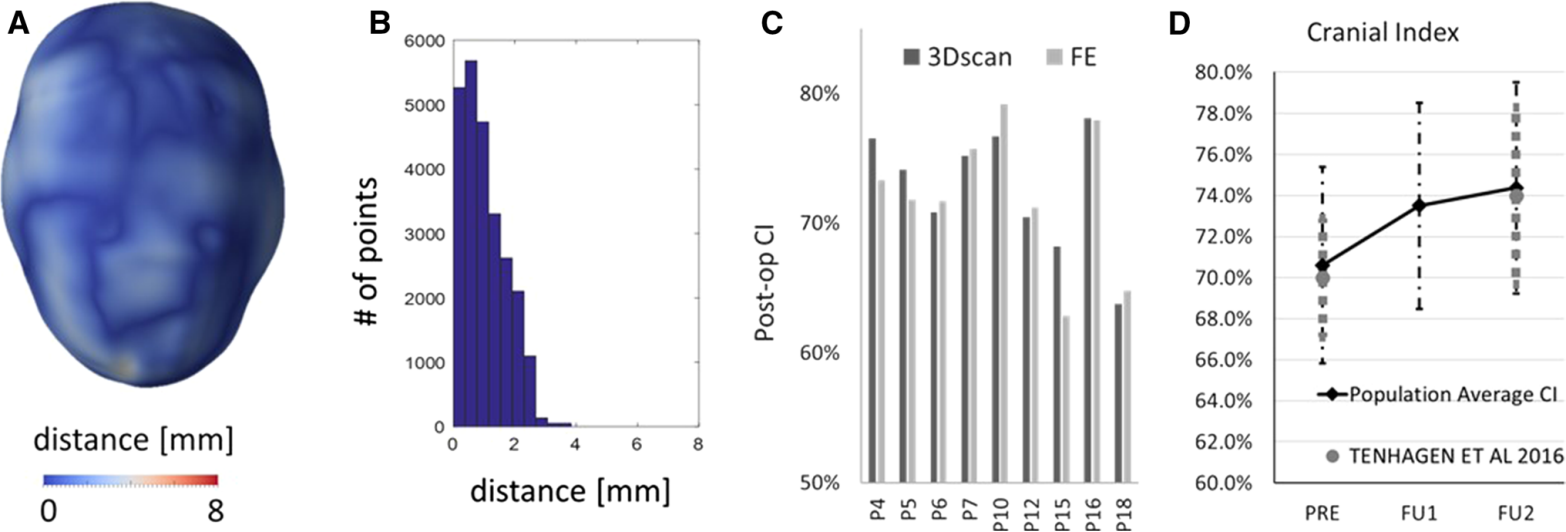
**a** Sample patient distance map between the predicted post-operative head shape for a representative patient. **b** Histogram showing the error distribution for the representative patient. **c** Bar chart showing a comparison between the post-op CI measured from 3D scan and that predicted from the FE simulations. **d** Population average CI measured from preoperative (PRE) models and simulated at the time of FU1 and FU2; in grey, the values of a similar population reported by Tenhagen et al. (2016)

The results of the comparison between the simulated post-explantation skull shape and the post-operative 3D scans visualized in terms of surface distance patterns ( Fig. 7a) and surface distance (error) distribution (Fig. 7b), demonstrated good shape matching between the simulated head shape and the on-table scan. Eight out of nine patients had over 80% of the error below 2 mm. The post-op CI was well predicted with a discrepancy of 1.9 ± 1.7% (Fig. 7c).

Figure 7d shows the change in the population average CI: average CI was 70.6% ± 4.8% at the time of insertion (PRE), 73.5% ± 5.0% at FU1 and 74.0% ± 4.3% at FU2. In grey are the values reported by Tenhagen et al. (2016) in an experimental study.

## Discussion

Surgical planning by means of computer simulations requires reliable tissue mechanics models: most studies in the literature use experimentally derived values of material properties to perform finite element analysis for surgical planning. More recently, the use of patient-specific material properties has shown great potential for the improvement of the capability of FEM to provide meaningful clinical information (Trabelsi et al. 2016; Zhang et al. 2017; Cosentino et al. 2019). Population-specific material properties have been extracted in recent studies (Knoops et al. 2018; Bosi et al. 2018) to overcome the complexity of non invasively retrieving patient-specific material properties while still allowing a high degree of accuracy and reliability: Bosi et al. (2018) found that the use of population-specific material properties for the modelling of transcatheter aortic valve replacement allowed for a decrease in simulated absolute expansion error from 5.3 to 2.5% as well as for a narrower distribution in prediction error. Knoops et al. (2018) used DoE to model inaccuracies in a population of patients undergoing orthognatic surgeries: the creation of an optimized material model for the face hard and soft tissues allowed for marked narrowing in prediction of cephalometric points movement, rendering the model more accurate in predicting facial change following maxillary movement.

In this paper, the methodology previously developed for the creation of a patient-specific anatomical model of SAC was expanded and extended to include more realistic material parameters for this specific patient population. Data in terms of spring expansion on-table and during follow-ups were used to tune the FE model for the SAC patient.

To tackle the difference in age between the head size at the time of CT anatomical acquisition and the time of SAC, a growth chart for the supraorbital calvarial volume was created using retrospective data from a cohort of untreated patients: the results showed a similar trend to that reported in the literature for intracranial volume growth of SC patients (Anderson et al. 2007). This methodology was tested and validated using available on-table 3D scans of patients from the cohort analysed: after linear rescaling the head models matched the respective 3D scan with an average error below the accepted threshold of 2 mm usually considered in maxillofacial surgery planning (Aung et al. 1995).

FE simulations of SAC using literature values for bone and suture mechanical properties provided acceptable results in terms of on-table spring opening, but suboptimally replicated spring opening at first and second follow-up. Therefore, a population-specific material model combination able to improve long-term prediction of spring performance was identified through a design of experiments approach. This showed that on-table spring opening was highly sensitive to the calvarial bone Young’s modulus value while the follow-up opening mainly depended on the relaxation modulus scale. Suture Young’s modulus had less effect on the opening results, as did the relaxation time scale, which describes the kinematics of expansion rather than the extent of expansion itself.

The model optimization yielded a marked improvement in prediction of spring opening at the time of FU1 and FU2, while the error in prediction for the on-table opening remained virtually the same compared to baseline values. The predicted population value for *E*_*B*_ (418 ± 217 MPa) was highly close to the value reported in the literature (421 MPa) for 6 month old children (Li et al. 2013) and used for the baseline model. The value of population Young’s modulus retrieved was also similar to that obtained in an experimental study carried out by our group (384 ± 133 MPa; Rodriguez-Florez et al. 2018)). The numerical model validation by means of comparison with post-op 3D scans shows good agreement between the shape predicted computationally and that retrieved on-table. Post-operative cranial index was predicted within 1.9% ± 1.7% for this group of patients: when comparing CI in the subpopulation used for testing the rescaling method, a similar error was found; therefore, it may be assumed that uncertainty in shape at the time of implantation affects also the final results.

This work shows a framework for producing and validating a predictive model of SAC in a SC patient population. The main limitation of this study is the limited numbers of simulated cases due to the lack of pre-operative CT images in routine SAC. A recently developed imaging technique—“black bone” MRI—has proved suitable for visualizing patent cranial sutures (Eley et al. 2014, 2017). The adoption of radiation free techniques for patient scanning may also dramatically increase the number of patients undergoing full head scan for pre- and long-term post-operative assessment, thus allowing for larger scale validation.

Additionally, long-term follow-up 3D images could provide insight into the capability of the model to predict long-term head reshaping after spring removal as a result of SAC (Beaumont et al. 2017; Knoops et al. 2017).

The quality of the material optimization is highly dependent on optimization data (spring measurements) as well as segmentation of the calvarial structures (skull and sutures). Although CT images provide an excellent definition for hard tissue (bone), segmentation of the cranial sutures had to be performed either through morphological operations or manually as suture material produces the same signal as the soft tissues (scalp and brain) during acquisition (Qian et al. 2015). Several groups in the past have attempted automatic reconstruction of cranial sutures using complex algorithms (Qian et al. 2015; Ghadimi et al. 2016), but such methodologies are still not widely available. All patients recruited for this study had a CT scan acquired in other centres prior to referral, and not repeated at GOSH before surgery to avoid additional radiations. Therefore, scanner systems as well as CT parameters varied slightly from one patient to another. The same grayscale values were used to segment each patient skull; however, as CT scans were acquired in different centres, slight differences in calibration between CT machines may have had an impact on the accuracy of the segmentation. It has been shown that clinical CTs are suitable for estimation of skull thickness (Lillie et al. 2014), and that Hounsfield Unit threshold levels for non-specific CT series can still be used for correctly detecting abnormal bone mineral density (Pickhardt et al. 2013).

A five point 3D surface scanner may allow in the future real-time acquisition of head shape for the creation of skull models and prediction of spring outcomes: if proven feasible, this would increase the number of patients with available 3D anatomical data collected at our centre before surgery, with improved acquisition consistency.

The present model is suitable for the prediction of SAC outcome in patients affected by sagittal craniosynostosis aged between 3 and 8 months (as the subgroup used for material optimization): adoption in different patient cohorts receiving spring cranioplasty for a different indication, such as posterior vault expansion (van Veelen and Mathijssen 2012) and correction of metopic (Lauritzen et al. 2008) and lambdoid (Arnaud et al. 2012) synostosis, may require a different material model due to the different nature of the pathology—syndromic craniosynostosis for patients affected by Crouzon syndrome and Apert syndromes who receive spring-assisted posterior vault expansion (de Jong et al. 2013)] or different age range (Davis et al. 2010; van Veelen et al. 2017): this methodology would still be valid if reproduced in a different patient cohort.

As mentioned in our previous paper, the main obstacle in predicting the final outcome of this procedure at the time of spring removal (which occurs 3–6 months after the insertion (Rodgers et al. 2017)) is the inability of the current linear model to predict calvarial growth. Recent studies have attempted to address such problem using numerical models for predicting calvarial growth in mice (Marghoub et al. 2018) and humans (Libby et al. 2017): assuming patients undergoing SAC follow normal growth trends, it may be possible to implement a similar model on the deformed model at the time of FU2 and simulate normal growth.

## Conclusion

In previous work, our group analysed biomechanical behaviour of cranioplasty springs (Borghi et al. 2017a, 2018) to understand the working mechanism of calvarial adaptation to spring distraction and loads experienced by the paediatric skull during reshaping. Recently, our group proved that spring cranioplasty can be modelled using finite element method to accurately predict head shape following spring insertion. We have hereby expanded this study to a larger population, which allowed fine tuning of the material model by identifying a material model combination able to improve prediction of spring performance during follow-up.

This model can now be used to perform prospective prediction of spring dynamics to inform surgical planning, perform distractor selection as well as improve pre-operative patient and parent information.
